# Imaging features of a brown tumor with extensive skull involvement: Relevance for dental radiology

**DOI:** 10.4317/jced.62709

**Published:** 2025-05-01

**Authors:** Artemisa Fernanda Moura Ferreira, Francisco de Assis Limeira-Júnior, José Jhenikártery Maia de Oliveira, Paulo Rogerio Ferreti Bonan, Marcelo Augusto Oliveira de Sales

**Affiliations:** 1 Postgraduate Program in Dentistry, Federal University of Paraiba, João Pessoa, Paraiba, Brazil; 2 Department of Morphology, Federal University of Paraiba, João Pessoa, Paraiba, Brazil; 3 Postgraduate Program in Dentistry, Federal University of Rio Grande do Norte, Natal, Rio Grande do Norte, Brazil

## Abstract

This case report describes a rare, asymptomatic brown tumor in a patient with end-stage renal disease. The lesion was incidentally detected during a computed tomography (CT) scan of the paranasal sinuses, performed upon a dentist’s recommendation to investigate maxillary sinusitis. CT imaging revealed an expansive osteolytic lesion with irregular margins and a ground-glass appearance involving the left side of the sphenoid and frontal sinuses. Subsequent magnetic resonance imaging (MRI) with multiplanar T1 and T2-weighted sequences without contrast demonstrated a solid tissue-like expansive lesion affecting the left frontal and sphenoid bones, mildly compressing adjacent cerebral parenchyma. Despite these findings, the patient remained asymptomatic. Conservative management, including pharmacological therapy with calcimimetics to control parathyroid hormone levels, was initiated. A follow-up MRI after five years showed lesion stability without significant changes. The patient later underwent a renal transplant, which effectively stabilized the bone disease and improved his quality of life. This case underscores the pivotal role of computed tomography (CT) in detecting incidental systemic skeletal changes and the indispensable importance of interdisciplinary collaboration in managing complex conditions in systemically compromised patients, where each professional’s expertise is crucial for the patient’s well-being.

** Key words:**Sinusitis, Brown Tumor, Hyperparathyroidism, Multidetector Computed Tomography, Magnetic Resonance Imaging, Multidisciplinary Care Teams.

## Introduction

Chronic kidney disease (CKD), particularly in its advanced stages, is frequently associated with secondary hyperparathyroidism (SHPT), leading to significant skeletal changes collectively known as CKD-mineral and bone disorder (CKD-MBD), which contribute substantially to patient morbidity and commonly involve multiple skeletal sites, with skull involvement being rare (1).

Brown tumors, severe manifestations of CKD-MBD, arise from excessive osteoclastic activity and fibroblast proliferation due to SHPT, with a reported prevalence of 1.5% to 1.75% among affected patients. Although often asymptomatic, such lesions can cause symptoms when near critical structures, leading to headaches, facial swelling, and ophthalmologic disturbances, including diplopia and proptosis (2).

Imaging is central to diagnosing these lesions, with computed tomography (CT) preferred for its ability to reveal expansile osteolytic defects with cortical thinning and well-defined margins. Magnetic resonance imaging (MRI) provides complementary information, typically showing low signal intensity on T1-weighted sequences and heterogeneous high signal intensity on T2-weighted sequences. Given their radiographic similarities, brown tumors must be distinguished from aneurysmal bone cysts, fibrous dysplasia, and central giant cell lesions, underscoring the need for thorough imaging assessment to prevent misdiagnosis and unnecessary surgical intervention (3).

This case report describes an extensive, asymptomatic brown tumor involving the skull base and frontal region in a CKD patient, incidentally identified during a CT scan of the paranasal sinuses.

Case Report

**Figure 1:**
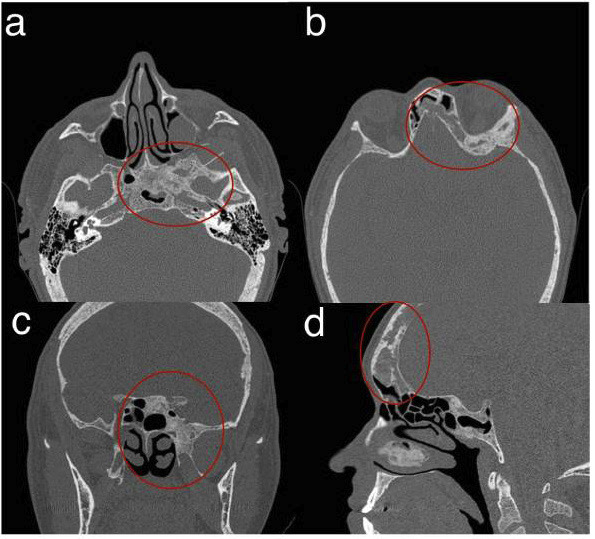
Computed Tomography Multi-slice: axial (a and b), coronal (c), and sagittal (d) scans showing hypodense images at the base of the skull and frontal region.

A 54-year-old male patient with a history of chronic kidney disease presented to his dentist with facial pain. According to the patient’s renal history and complaints, the dentist recommended a CT scan of the paranasal sinuses (multi-slice 64, with 1-mm thickness and 1-mm reconstruction interval) to assess for potential maxillary sinus pathology. The scan confirmed maxillary sinusitis and incidentally revealed an expansive osteolytic lesion with irregular margins and a ground-glass appearance involving the left aspects of the sphenoid and frontal sinuses (Fig. 1a-d). Clinical and radiographic evaluations indicated a brown tumor, a non-neoplastic reactive lesion caused by excessive osteoclastic bone resorption associated with secondary hyperparathyroidism.

**Figure 2:**
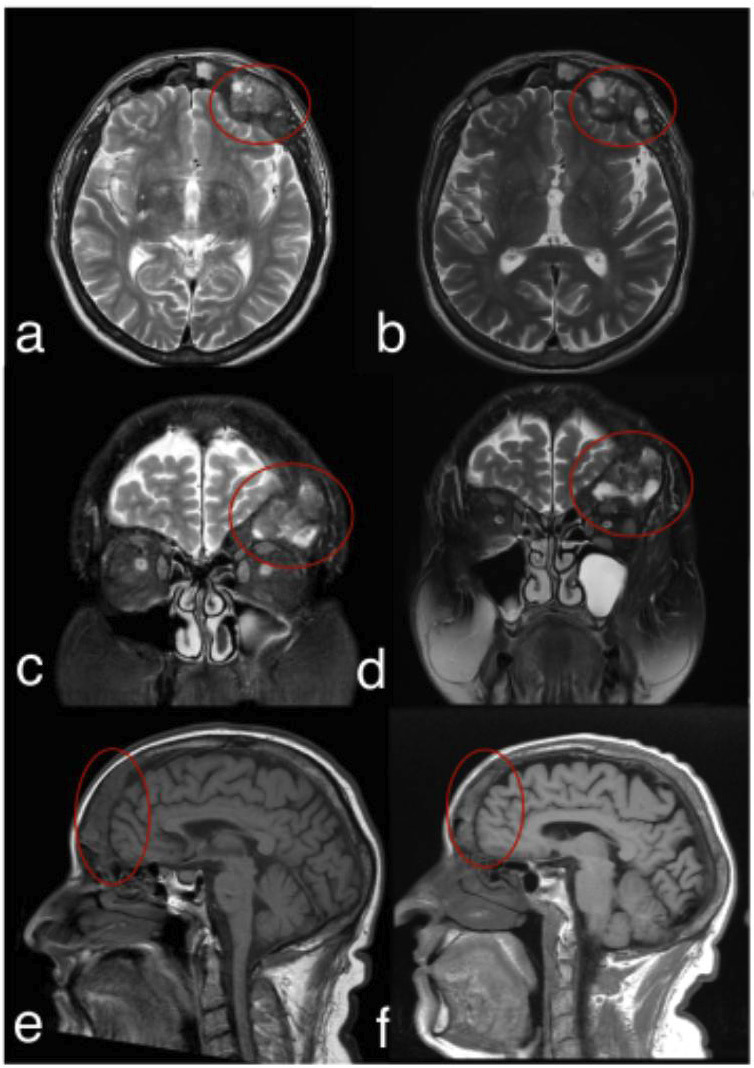
Magnetic resonance images showing the initial presentation (a, c, e) and the stable appearance of the lesion after five years of follow-up (b, d, f). T2-weighted sequences in axial and coronal views (a–d) and T1-weighted sequences in the sagittal plane (e–f) illustrate a lesion affecting the skull base and frontal region.

Upon reviewing the CT findings, the patient’s nephrologist, who managed his hemodialysis treatment, ordered an MRI to assess further the lesion’s extent and impact on adjacent structures. The MRI, performed with multiplanar T1- and T2-weighted sequences without contrast, revealed an expansile lesion with solid tissue characteristics affecting both frontal bones, with greater involvement of the left paramedian region. The lesion exerted a mild mass effect on the adjacent cerebral parenchyma. Despite these findings, the patient remained asymptomatic throughout the evaluation period. The nephrologist opted for conservative management, including pharmacological therapy with calcimimetics to regulate parathyroid hormone levels. The patient was closely monitored, and a follow-up MRI five years later demonstrated lesion stability with no significant changes in size (Fig. 2a-f).

Subsequently, the patient underwent a renal transplant, which stabilized the bone disease and significantly improved his quality of life. This case highlights the critical role of dental professionals in managing patients with chronic kidney disease. Through routine examinations and imaging, dentists can detect incidental findings suggestive of systemic conditions, enabling early diagnosis and timely intervention. This proactive approach facilitates timely management, reduces morbidity, and may prevent the need for more invasive treatments.

This study was approved by the local Institutional Research Board (protocol 83302224.9.0000.5176). The patient provided written informed consent to publish this case report and accompanying images.

## Discussion

Brown tumors are rare, non-neoplastic lesions resulting from increased osteoclastic activity due to hyperparathyroidism. While they typically affect bones like the ribs, clavicles, and pelvis, cranial involvement is rare (4). In this case, CT imaging revealed direct involvement of the frontal and sphenoid bones, an uncommon presentation that underscores the exceptional nature of this diagnosis. Although a thorough understanding of skull anatomy is crucial in dental practice, many dentists have limited experience in interpreting tomographic images of the skull, which is considered an anatomically complex area (5).

Brown tumors are generally asymptomatic; however, large lesions may cause complications. When located at the skull base, they can lead to neurological symptoms due to the involvement of the central nervous system (1). In this case, the patient remained asymptomatic despite the lesion’s size and proximity to major anatomical structures such as the orbit, frontal sinus, and brain.

Brown tumors are infrequently detected in the craniofacial region, so their identification through imaging is particularly significant. As patients undergoing long-term hemodialysis are at increased risk of developing skeletal alterations, dental professionals must be vigilant in recognizing craniofacial imaging characteristics indicative of systemic disorders and understanding their clinical implications (6). In some cases, CT findings may be the first indication of an undiagnosed systemic condition with potentially severe complications. This highlights the importance of thoroughly analyzing the entire tomographic volume, regardless of the exam’s initial clinical indication.

In this case, the incidental discovery of a rare skull-based brown tumor prompted a change in medical management, leading to a pharmacological approach aimed at stabilizing the lesion and preventing further complications (7). Imaging’s ability to reveal asymptomatic skeletal lesions in systemically compromised patients underscores its value in interdisciplinary healthcare (8).

Brown tumors can be challenging to diagnose, as their clinical presentation and imaging features may closely resemble those of other bone lesions, potentially delaying appropriate management. Sometimes, differentiation between brown tumors and central giant cell lesions remains difficult, even after histopathologic analysis (9). In this case, the patient’s clinical history and laboratory findings were crucial in establishing the correct diagnosis.

Long-term follow-up demonstrated that effective systemic management stabilized the lesion, aligning with findings reported in previous studies (4,10). This case reinforces the pivotal role of dental and maxillofacial radiology in identifying rare systemic skeletal manifestations early, ensuring proper diagnosis, and guiding appropriate clinical decisions. The collaboration between dental and medical professionals highlights the importance of a multidisciplinary approach to managing complex health conditions and providing comprehensive patient care.

## Conclusions

This case highlights the importance of computed tomography (CT) in the early diagnosis of rare skeletal lesions in patients with chronic kidney disease (CKD). The incidental identification of such a lesion during a dental evaluation emphasizes dentistry’s essential role in detecting asymptomatic changes in systemically compromised patients.

## Data Availability

None.
